# Recent Progress on One-Pot Multisubstrate Screening

**DOI:** 10.1021/acs.oprd.3c00128

**Published:** 2023-07-11

**Authors:** Matisyahu S. Fogel, Kazunori Koide

**Affiliations:** Department of Chemistry, University of Pittsburgh 219 Parkman Avenue, Pittsburgh, Pennsylvania 15260, United States

**Keywords:** high-throughput screening, enantioselectivity, diastereoselectivity, gas chromatography, liquid chromatography, mass spectrometry

## Abstract

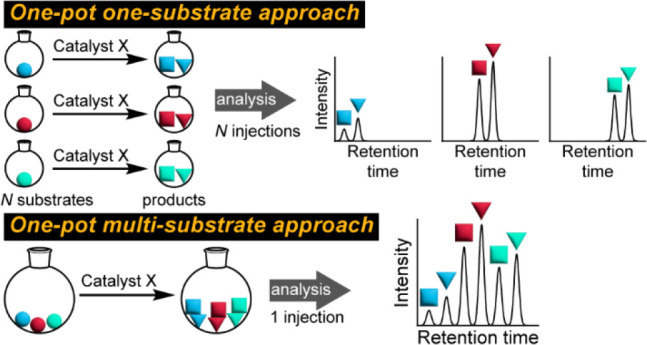

Traditionally, new synthetic reactions have been developed using a model substrate to screen reaction conditions before testing the optimized conditions with a range of more complex substrates. In 1998, Gao and Kagan pooled multiple substrates in one pot to study the generality of an enantioselective method. Although such one-pot multisubstrate screenings may be powerful, few applications have appeared in the literature. With the advancement of various chromatography techniques, it may be time to revisit this underutilized platform. This review article discusses the applications of one-pot multisubstrate screenings as a method for developing new synthetic methods.

Synthetic methodologies require high efficiency, reproducibility, and generality. Research from the initial discovery of a new reaction to its improvement to meet these criteria demands numerous experiments. Historically, synthetic technologies were optimized through repetitive trial and error in a one-at-a-time fashion. To expedite this process, high-throughput experimentation (HTE) has become a viable alternative to the traditional approach (Figure 1). The first parallel HTE was reported in 1996 by the Burgess group in a 96-well format.^1^ Today, multiwells (96, 384, or 1536 wells) are used as a platform for HTE.^1−12^ Recent work using HTE has provided significant chemical insights and led to more ideal synthetic methodologies. However, HTE requires specialized and expensive infrastructure, and when using 384 or 1536 wells, it is problematic to use volatile solvents (due to solvent evaporation at microliter volumes) or heterogeneous conditions (e.g., Pd/C, alkali metal, etc. due to difficulty in measuring out nanomolar amounts of solvent reproducibly and accurately). Additionally, the analysis of hundreds to thousands of reaction wells remains a bottleneck in HTE even if ultraperformance liquid chromatography (UPLC) is available.

**Figure 1:**
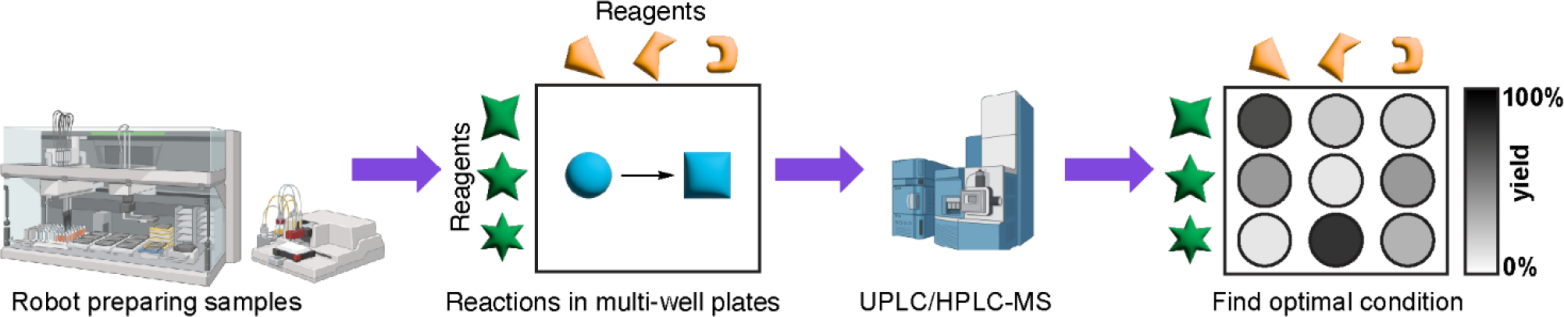
An example of a typical HTE workflow. Robots dispense reagents into multiwell plates. After the reaction, UPLC/HPLC-MS analysis is used to find the optimal condition.

An alternative to HTE was developed in 1998 by Gao and Kagan, which they termed “one-pot multi-substrate screening (OPMSS).” The premise of OPMSS is that multiple parallel reactions in one pot generate a set of data upon analysis of the mixture. This produces many data points with a relatively small number of experiments. Indeed, they stated that “*the most useful approach in the fast screening of a new asymmetric catalyst is to get a set of information from one experiment*” and that “*the easiness and simplicity of this method make it very convenient for a preliminary evaluation of a chiral reagent or catalyst*.”^13^ Gao and Kagan proposed that OPMSS could be a viable tool to determine the enantioselectivity of reactions. They demonstrated this by applying OPMSS to the asymmetric reduction of a set of ketones using a Corey catalyst. They combined multiple ketones in the same flask, reduced them, and then analyzed the reaction mixture using chiral HPLC to find the enantiomeric excess (ee) of each alcohol product. They compared the ee of each alcohol when the reduction was performed as a mixture of ketones to the ee when each ketone was reduced individually to ensure no interference or induction by the presence of other substrates. The verification that there is no interference between substrates is essential (Figure 2). As Kagan wrote in a 2005 review article, “*the information extracted from the one-pot multi-substrate screening will be valid only if there are no interactions between the products and the catalysts or reagents*.”^14^ They reduced each ketone as part of different substrate sets to ensure that different types of substrates were orthogonal with respect to the reduction enantioselectivity. For the first set of ketones, one run of chiral HPLC was able to resolve substrates **1a**–**d** and products **2a**–**d** (Scheme 1). However, for the other, larger sets of ketones, direct HPLC analysis was impossible because of peak overlap, and additional flash column chromatography was necessary to separate the compounds into three fractions, which were then analyzed by chiral HPLC.^13^ After Gao and Kagan introduced OPMSS as a feasible method, multiple groups used the platform to develop stereoselective asymmetric catalysis methodologies. Satyanarayana and Kagan reviewed the applications of OPMSS in 2005.^14^ This review will briefly discuss the papers already summarized in the 2005 review article and mainly focus on examples of OPMSS published after their review.

**Figure 2:**
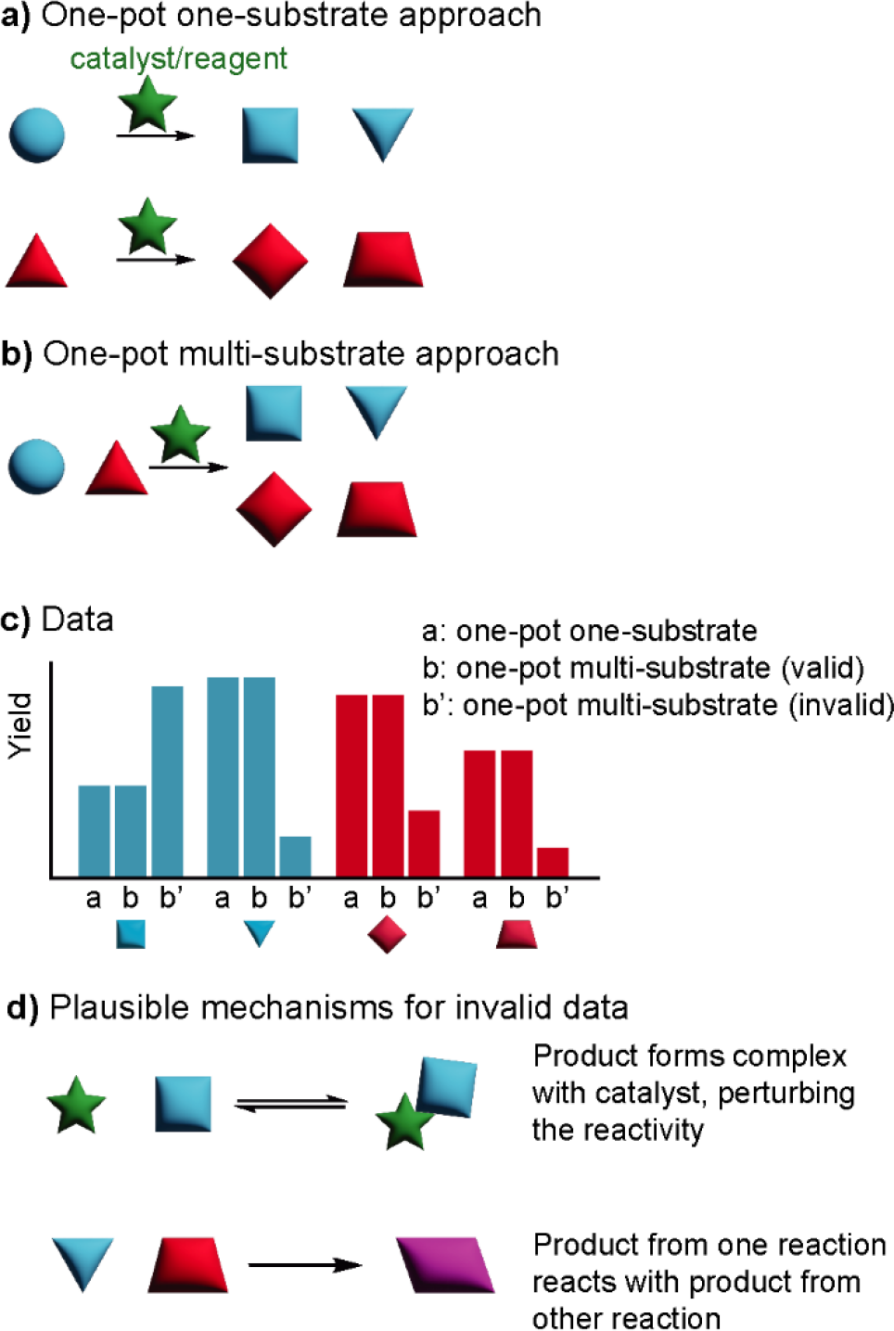
To use multisubstrate screening, the verification of no interference between substrates is crucial. (a) In the traditional approach, multiple reactions with different substrates are performed separately, (b) while in the one-pot multisubstrate approach, multiple substrates are pooled in the same flask. (c) Control experiments are important to verify that data obtained from one-pot multisubstrate reactions are the same as in single-substrate reactions. This is done by performing single-substrate reactions and comparing the results to that of multisubstrate reactions. If the results are consistent, then OPMSS is a valid method to apply to the system being investigated. (d) Two forms of interference that can cause one-pot multisubstrate data to differ from single-substrate reactions.

**Scheme 1:**
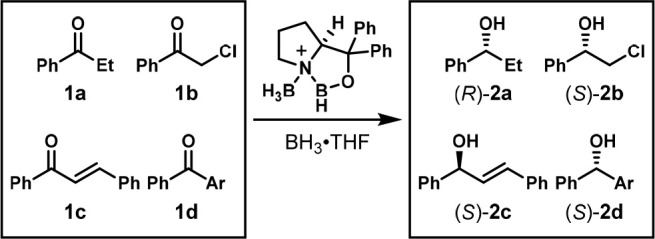
One-Pot Enantioselective Reduction of Four Ketones Reaction conditions: solution of ketones **1a**–**d** (2 mmol total), diphenylprolinol (0.1 mmol), BH_3_ (2.1 mmol), and THF (4.1 mL, 0.5 M) for 1 h under argon. Ar = 4-CF_3_Ph.

In 1998, the Gennari group also used the same approach and combined four substrates to screen chiral catalysts for the nucleophilic addition of diethylzinc to aldehydes **3a**–**d** to form alcohols **4a**–**d** (Scheme 2).^15^ However, they did not show that the one-pot multisubstrate reaction did not suffer from interference between substrates. The enantiomeric ratios were determined by chiral gas chromatography (GC).

**Scheme 2:**
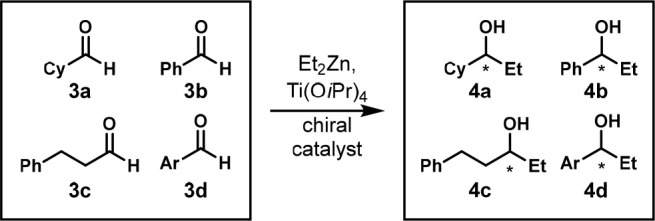
One-Pot Enantioselective Nucleophilic Addition of Diethylzinc to Four Aldehydes Reaction conditions: mixture of aldehydes **3a**–**d** (0.025 mmol each, 0.1 mmol total), Ti(O*i*Pr)_4_ (0.12 mmol), ligand (0.004 mmol), Et_2_Zn (0.22 mmol), −20 °C, 16 h. Cy = cyclohexyl, Ar = 4-ClPh.

In 2000, the Liskamp group prepared a library of chiral ligands on a solid support and mixed four substrates in a reaction mixture. They performed a similar addition of diethylzinc to aldehydes **3a**, **3b**, **3d**, and **3e** to form alcohols **4a**, **4b**, **4d**, and **4e** (Scheme 3). Chiral GC was used to analyze the mixtures of products. A gas chromatogram (Figure 3) indicates the separation might not have been proactively considered, although they mentioned that GC retention times were important parameters.^16^ Testing substrates individually to validate their experiments was not reported.

**Scheme 3:**
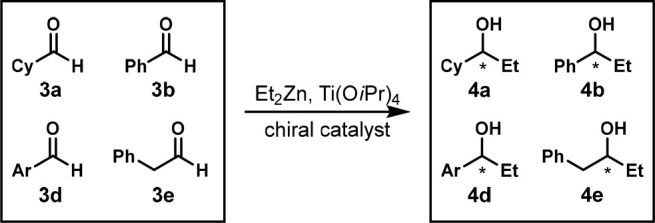
One-Pot Enantioselective Nucleophilic Addition of Diethylzinc to Four Aldehydes Reaction conditions: mixture of aldehydes **3a**, **3b**, **3d**, and **3e** (0.0125 mmol each), Ti(O*i*Pr)_4_ (0.06 mmol), ligand (0.002 mmol), Et_2_Zn (0.110 mmol), −20 °C, overnight, under Ar. Cy = cyclohexyl, Ar = 4-ClPh.

**Figure 3:**
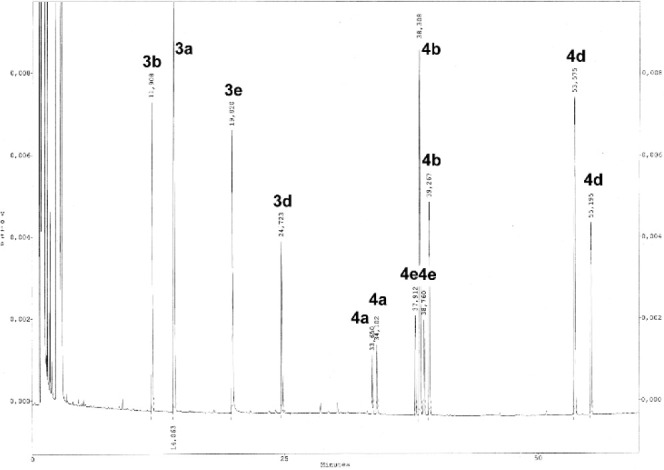
Gas chromatogram of a mixture of the reaction shown in Scheme 3. Modified with permission from ref (16). Copyright 2000 American Chemical Society.

In 2001, the Piarulli and Gennari group used the same screening platform to screen different chiral ligands for the conjugate addition of diethylzinc to nitroalkenes **5a** and **5b** to form **6a** and **6b** (Scheme 4).^17^ The potential interference was not studied.

**Scheme 4:**
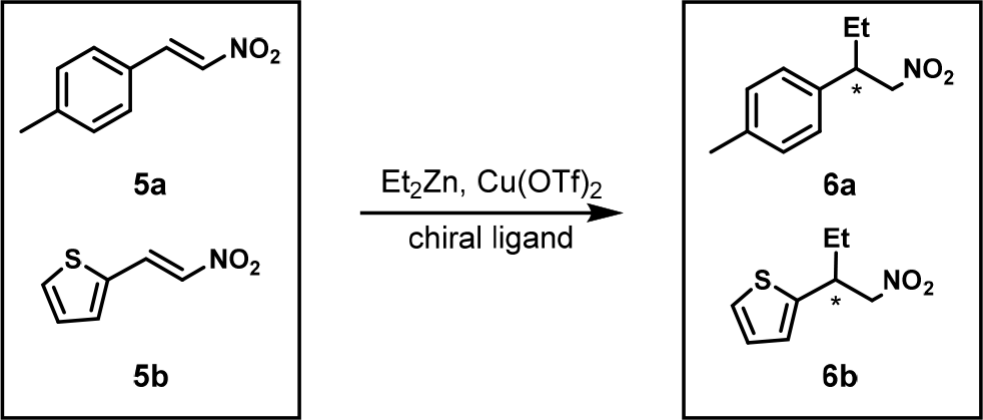
One-Pot Enantioselective Conjugate Addition of Diethylzinc to Two Nitroalkenes Reaction conditions: **5a** (0.10 mmol), **6a** (0.10 mmol), ligand (0.011 mmol), Cu(OTf)_2_ (0.010 mmol), −20 °C, 3 h.

In 2002, the Wolf group screened enantioselective catalysts for the nucleophilic addition of an ethyl group to aldehydes.^18^ This is the same transformation as the Liskamp system. The authors mixed three substrates, **3a**, **3b**, and **3f**, to form products (*R*)-**4a**, (*R*)-**4b**, and (*R*)-**4f** (Scheme 5) and used chiral GC for analysis (Figure 4).

**Scheme 5:**
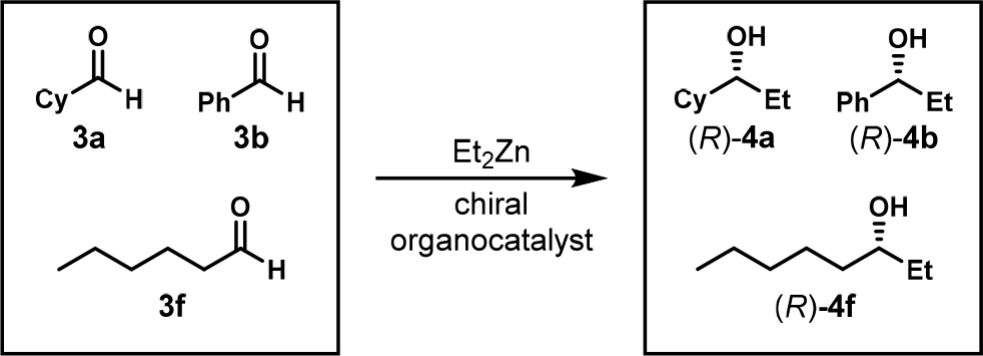
One-Pot Organocatalyzed Enantioselective Addition of Diethylzinc to Three Aldehydes Reaction conditions: mixture of aldehydes **3a**, **3b**, and **3f** (0.47 mmol total), catalyst (0.04 mol), Et_2_Zn (1.1 mmol), hexanes (3.1 mL), 0 °C, 16 h. Cy = cyclohexyl.

**Figure 4:**
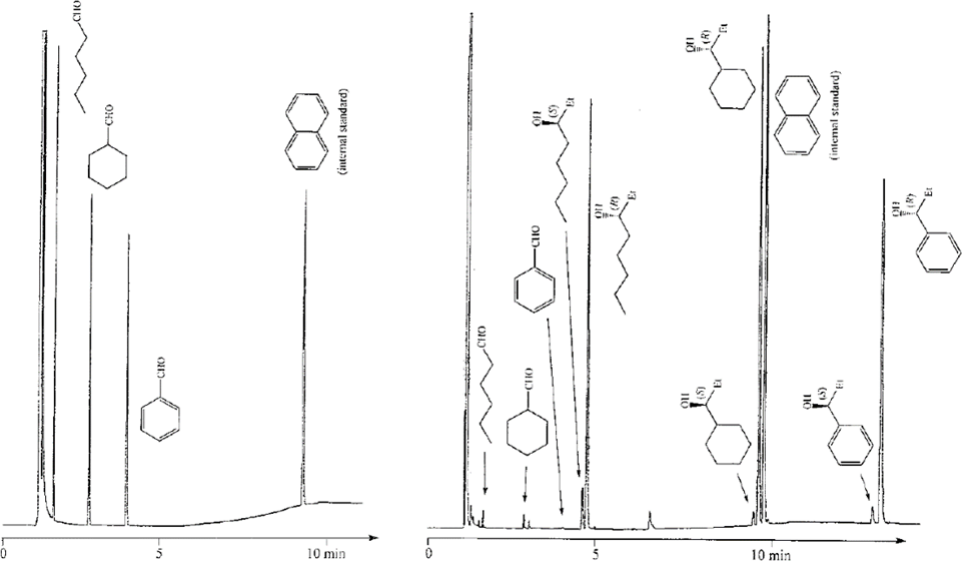
Gas chromatogram of starting materials and internal standard for three-substrate mixture (left) and product mixture (right). Reproduced with permission from ref (18). Copyright 2002 American Chemical Society.

In 2002, the Feringa group developed an enantioselective conjugate addition of diethylzinc to nitroalkenes. Nine aromatic substrates **5a**–**i** were pooled into one pot to screen for optimal catalysts to form **6a**–**i** (Scheme 6).^19^ Two criteria were articulated: (1) the product peaks should not overlap in the chromatogram and (2) the different substrates and products should not interfere with each other during the reaction. With two optimal catalysts, four aliphatic substrates were tested together to study the generality of the two catalysts.

**Scheme 6:**
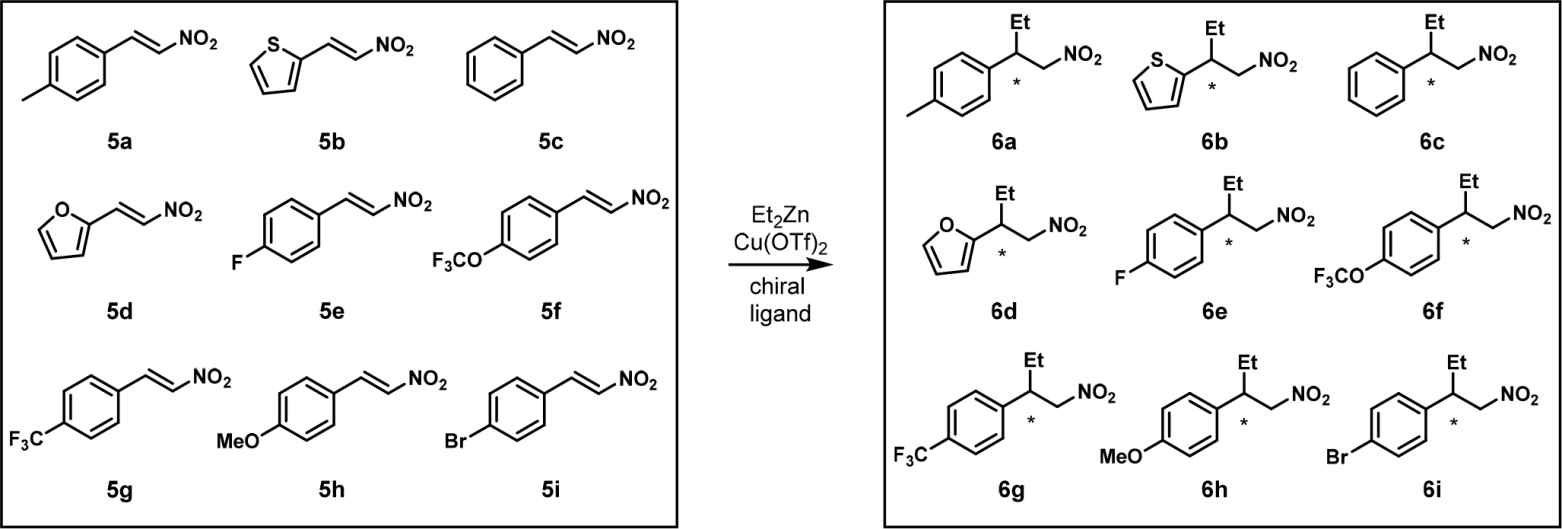
One-Pot Enantioselective Addition of Diethylzinc to Nine Aryl Nitroalkenes Reaction conditions: mixture of nitroalkenes **5a-i** (0.25 mmol each), Cu(OTf)_2_ (0.045 mmol), ligand (0.09 mmol), Et_2_Zn (3.4 mmol), toluene (3 mL), hexanes (3.4 mL), −45 °C, 3 h.

In 2004, Goddard and Reymond pooled 20 substrates to determine the substrate scope for enzyme-catalyzed hydrolysis of esters **7** to carboxylic acid **8** and 1,2-diols **9** (Scheme 7).^20^ They used reverse-phase HPLC with different wavelengths to analyze the data and determine reactivity (Figure 5).

**Scheme 7:**
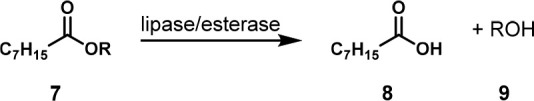
One-Pot Enzyme-Catalyzed Hydrolysis of 20 Octanoyl Esters Reaction conditions: enzyme solution (25 μg/mL), pH 7.4 aq phosphate buffer (50 μL) containing BSA (1 mg/mL), DMF (20% v/v), mixture of esters **7** (0.2 mM total), 25 °C, 1 h. R = hydroxy-substituted alkyl group with UV-active tag.

**Figure 5:**
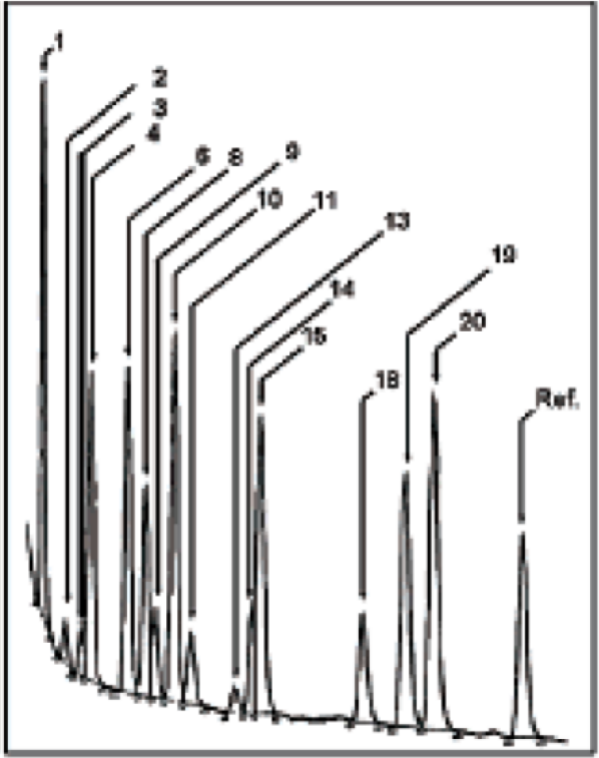
HPLC chromatogram of mixture of 20 diol products produced from enzyme-catalyzed ester hydrolysis. Reproduced with permission from ref (20). Copyright 2004 American Chemical Society.

In 2005, the Pfaltz group applied OPMSS to the iridium-catalyzed enantioselective hydrogenation of alkenes. They hydrogenated four terminal alkenes **10a**–**d** in one pot using an iridium catalyst and H_2_ bubbled into the flask (Scheme 8). They used chiral GC to analyze the reaction mixture, thereby successfully resolving all enantiomers of products **11a**–**d** (Figure 6). They also demonstrated no interference by other substrates, as they measured the ee values of the products in single-substrate reactions, which corroborated the values in the multisubstrate experiment.^21^

**Scheme 8:**
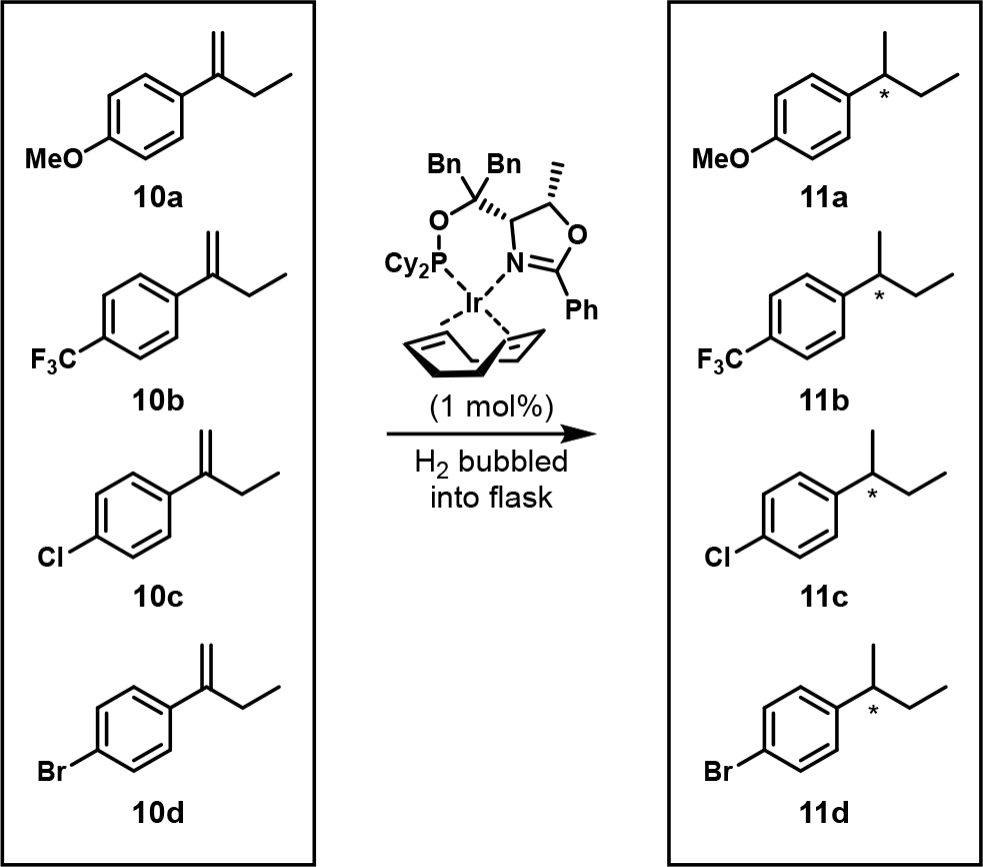
One-Pot Iridium-Catalyzed Enantioselective Hydrogenation of Four Alkenes The detailed reaction conditions are unavailable.

**Figure 6:**
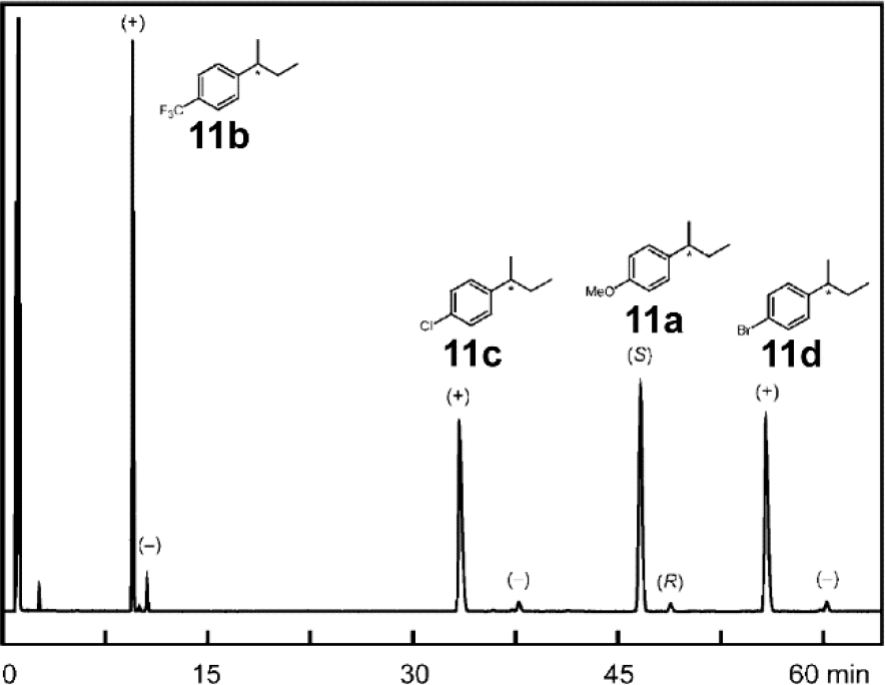
Gas chromatogram of separated enantiomers of products **11a**–**d**. Reproduced with permission from ref (21). Copyright 2005 WILEY-VCH Verlag GmbH & Co. KGaA, Weinheim.

In 2005, the Feringa group employed OPMSS for the enantioselective hydrogenation of acyclic enamides **12** to amides **13** using a Rh catalyst with a chiral phosphoramidite ligand (Scheme 9).^100^ They first demonstrated that the reactions were orthogonal by verifying that the ee values of single-substrate and one-pot multisubstrate reactions were identical for five substrates. They then showed that up to eight enamides and their reduced products could be resolved by chiral GC (Figure 7).

**Scheme 9:**
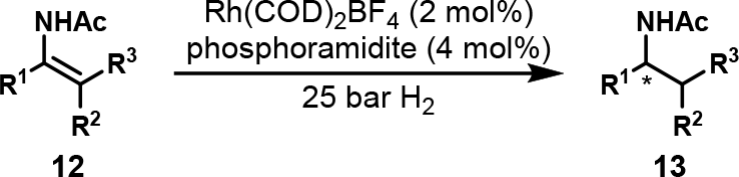
General Structure of Pooled Enamides That Underwent Rh-Catalyzed Hydrogenation The detailed reaction conditions are unavailable.

**Figure 7:**
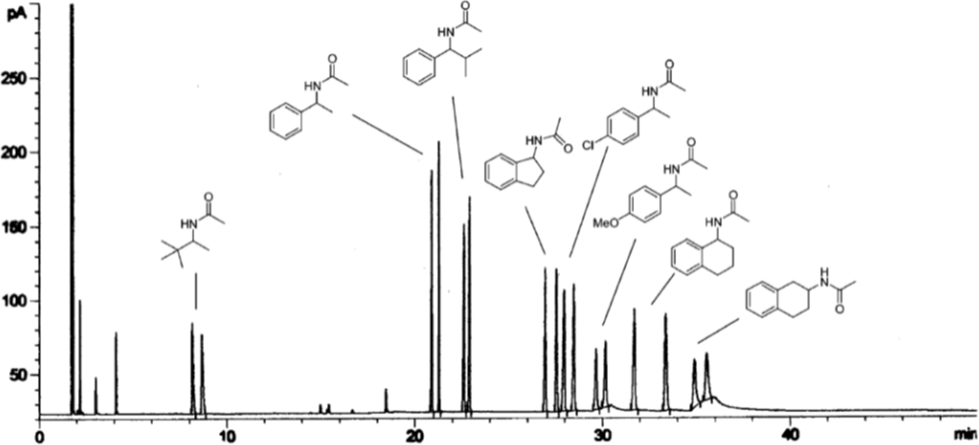
Racemic product mixture of hydrogenation products of eight enamides. Reproduced with permission from ref (100). Copyright 2005 American Chemical Society.

In 2006, the Kempe group developed a metal-catalyzed enantioselective hydrosilylation of ketones **14a**–**g** to form products **15a**–**g** using OPMSS (Scheme 10). They screened the reactivity of three sets of three or four ketones with five silanes (diphenylsilane, triethylsilane, pentamethyldisiloxane, methylphenylsilane, and dimethylphenylsilane) and six metal salts {[Ni(COD)_2_], [(DME)NiCl_2_], [(COD)PdCl_2_], [(COD)IrCl]_2_, [(COD)RhCl]_2_, and [(COD)RuCl_2_]} and used DIOP as a chiral ligand. They used chiral GC to determine percent conversions and ee values (Figure 8). Control experiments were not reported to confirm the orthogonality of the substrates. Using this screening protocol, they discovered that the Ni(0) catalyst and diphenylsilane reduced acetophenone **14a** with the highest ee, while the Rh(I) catalyst with methylphenylsilane afforded the highest ee for *tert*-butyl phenyl ketone **14d**. This result highlights the importance of OPMSS, as Ni(0) catalyst systems were previously unknown to display this reactivity. It also shows that when optimizing a reaction using a “model substrate,” it is possible to miss specific substrates that require different conditions.^22^

**Scheme 10:**
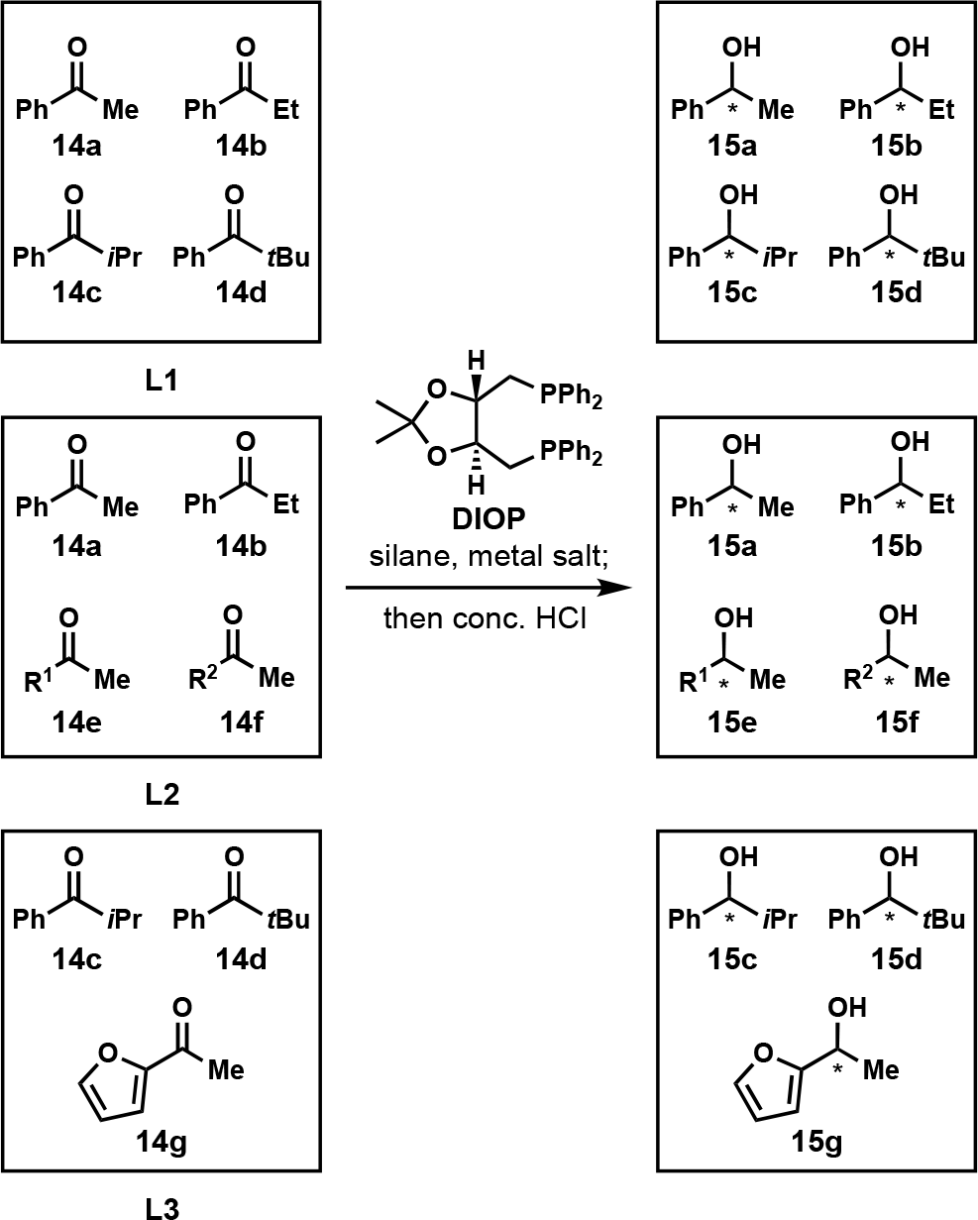
One-Pot Enantioselective Reduction of Three Libraries of Ketones (L1, L2, and L3) Using a Metal Salt, Silane and DIOP as a Chiral Ligand Metal Salts: {[Ni(COD)_2_], [(DME)NiCl_2_], [(COD)PdCl_2_], [(COD)IrCl]_2_, [(COD)RhCl]_2_, or [(COD)RuCl_2_]}. Silanes: Diphenylsilane, Triethylsilane, Pentamethyldisiloxane, Methylphenylsilane, or Dimethylphenylsilane. Reaction conditions: library of ketones (0.5 mmol total), metal salt (0.02 mmol), DIOP (0.02 mmol), silane (2.4 mmol), dodecane (internal standard, 0.5 mmol), toluene (2.5 mL), room temperature, 22 h. R^1^ = 4-ClPh, R^2^ = 4-FPh.

**Figure 8:**
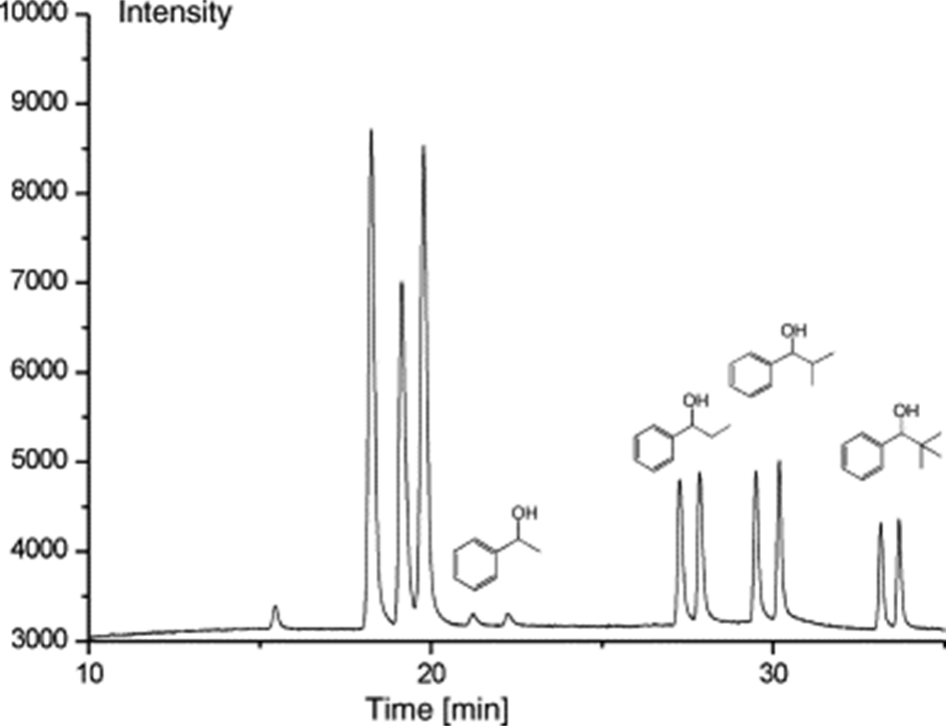
Gas chromatogram of alcohols **15a**–**d**. Reproduced with permission from ref (22). Copyright 2006 Elsevier.

In 2008, the Laschat group reported the enantioselective reduction of aryl ketones using α-pinene-derived aminoalcohols and BH_3_ (Scheme 11). Chiral GC was used to analyze a mixture of four aryl ketones **14a**–**c** and **14h** and the reduced products **15a**–**c** and **15h** (Figure 9). They found that only one of their aminoalcohols, **16**, provided good enantioselectivity and that adding trimethyl borate increased the ee. Individual experiments were not reported to confirm the lack of interference.^23^

**Scheme 11:**
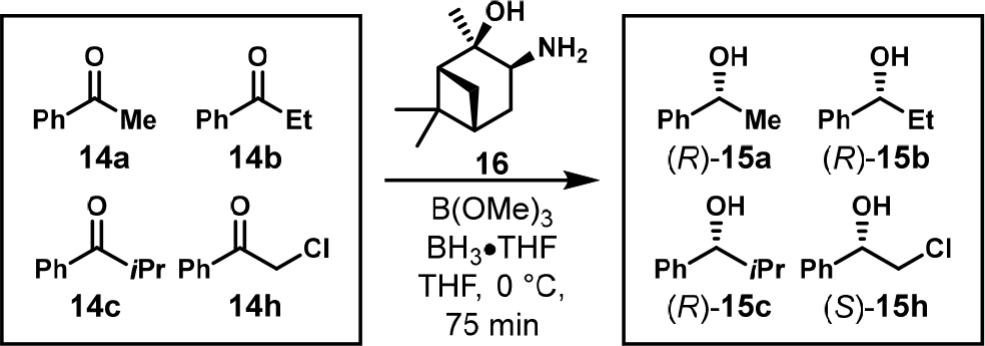
One-Pot Enantioseletctive Reduction of Four Aryl Ketones with Aminoalcohol **16** Reaction conditions: mixture of ketones (0.25 mmol each), B(OMe)_3_ (0.1 mmol), BH_3_·THF (1 mmol), **16** (0.1 mmol), THF (4 mL), 0 °C to room temperature, 75 min.

**Figure 9:**
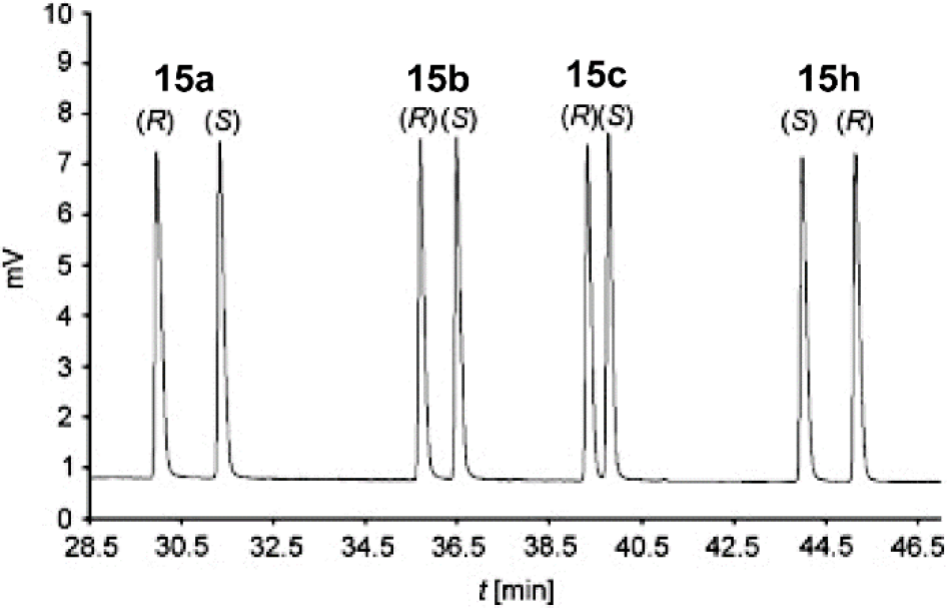
Gas chromatogram showing the separation of a racemic mixture of four alcohol reduction products. Modified with permission from ref (23). Copyright 2008 Elsevier.

In 2008, the Collin and Zouioueche groups described the enantioselective reduction of aryl ketones in water using a ruthenium catalyst. Chiral GC was used to analyze a mixture of the reduction of six aryl ketones, **14a** and **14h**–**l**, and their products, **15a** and **15h**–**l** (Figure 10a). As a control experiment, they reduced **14l** as a single substrate and in the presence of five other ketones. The control experiment showed that the presence of other substrates increased the ee from 55% to 68%, thereby indicating that there was induction by the other substrates. Nevertheless, they performed a multisubstrate screening of the six ketones with eight different ligands and then a different set of six ketones, **14a**, **14e**, **14h**, **14j**, **14l**, and **14m**, with 12 ligands to produce products **15a**, **15e**, **15h**, **15j**, **15l**, and **15m** (Figure 10b). Ligand **17** was identified to produce the highest ee values for *ortho*-substituted phenyl ketones, while all other ketones garnered better enantioselectivity with ligand **18** (Scheme 12). They then tested eight different ketones in single-substrate reactions to compare the ee using **17** and **18** and showed the trend that provided superior ee values.^24^

**Figure 10:**
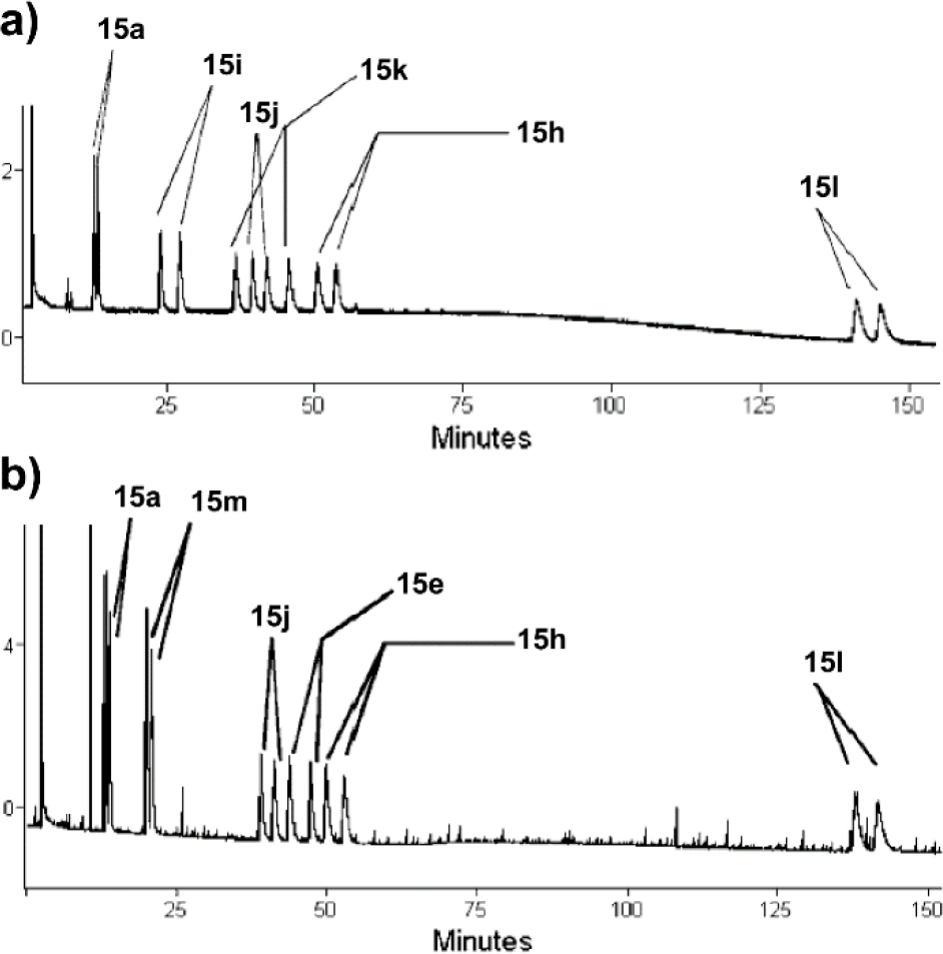
(a) Gas chromatogram of products of the first set of ketones using a chiral column. (b) Gas chromatogram of products of the second set of ketones using a chiral column. Modified with permission from ref (24). Copyright 2008 WILEY-VCH Verlag GmbH & Co. KGaA, Weinheim.

**Scheme 12:**
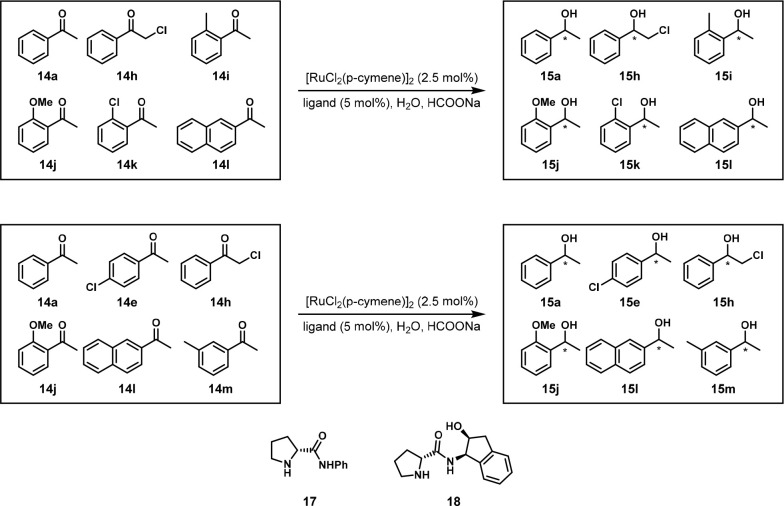
Two Sets of Aryl Ketones Were Enantioselectivity Reduced Using One-Pot Multisubstrate Screening with a Ruthenium Catalyst and Chiral Ligand in Water Reaction conditions: ketone mixture (0.5 mmol each), [RuCl_2_(*p*-cymene)]_2_ (0.075 mmol), ligand (0.15 mmol), HCOONa (10 mmol), H_2_O (4 mL), 30 °C. Ligands **17** and **18** produced the highest enantioselectivities depending on the ketone.

In 2010, the Fiaud group reported an enzyme-catalyzed kinetic resolution of racemic secondary alcohols through acylation. They examined the resolution of eight racemic alcohols *rac*-**19a**–**h** catalyzed by the lipase enzyme *CAL-*B (Figure 11). They reacted a racemic mixture of the alcohol with succinic anhydride in the presence of *CAL*-B lipase and then treated the reaction mixture with Na_2_CO_3_. Only one enantiomer was reactive with the lipase, so the unreactive enantiomer was recovered upon biphasic extraction with diethyl ether. The acylated alcohol was captured in the aqueous phase as the sodium carboxylate. The succinyl ester was subsequently saponified using NaOH to afford the enantiomerically enriched alcohol. They tested each substrate individually in a one-pot two-substrate mixture and in a one-pot four-substrate mixture and determined the enantioselectivity (*E*) using chiral HPLC. They found that, in two-substrate mixtures, pooling a reactive and nonreactive substrate provides similar *E* values to the individual reactions. However, in mixtures where both substrates were reactive, the % conversion was reduced, which the authors suggested might be due to the “imprinting” of the enzyme by a reactive substrate, which reduced the reactivity of the other substrate. Nevertheless, in one-pot two-substrate screens, the enantioselectivity was still similar to the individual reactions. In one-pot four-substrate screens, many substrates showed significantly lower ee values of the unreactive isomer than in individual screening because of a lower percent conversion (Table 3 of their paper). Additionally, some substrates (**19c**, **19e**, and **19f**) showed different ee values for the reactive isomer in OPMSS and individual screening (Table 1). This is the second example of applying OPMSS to enzyme catalysis.^25^

**Figure 11:**
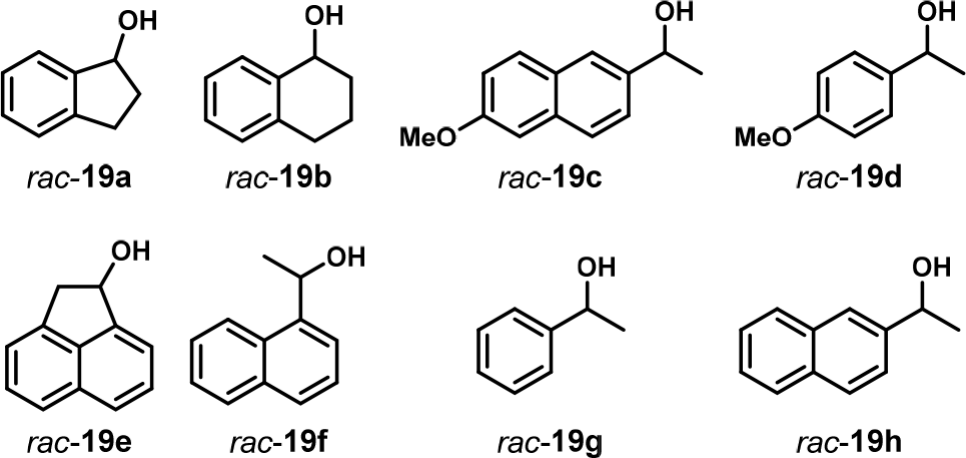
Eight racemic alcohols underwent kinetic resolution by *CAL-B* lipase and succinic anhydride.

**Table 1:** Reactive Alcohol Isomer ee Values That Had Divergent Values in Individual- and Four-Substrate Reactions

substrate	individual (% ee)	OPMSS (% ee)
**19c**	99	79
**19e**	36	90
**19f**	91	61

In 2011, the Collin and Zouioueche groups reported an OPMSS for reducing aliphatic ketones using chiral GC (Figure 12). They mixed seven ketones **20a**–**g** and reduced them to the corresponding alcohols **21a**–**g** using a ruthenium catalyst in water (Scheme 13). They demonstrated that with ligand **17**, the chiral alcohol products of the seven ketones had very similar ee values in single- and multisubstrate screenings. They then reduced those seven ketones with 11 different ligands and found that “*enantioselectivity depends both on the nature of ligand and substrate*.” The results of the multisubstrate screen were applicable to other ketones with similar properties to those in the OPMSS.^26^

**Figure 12:**
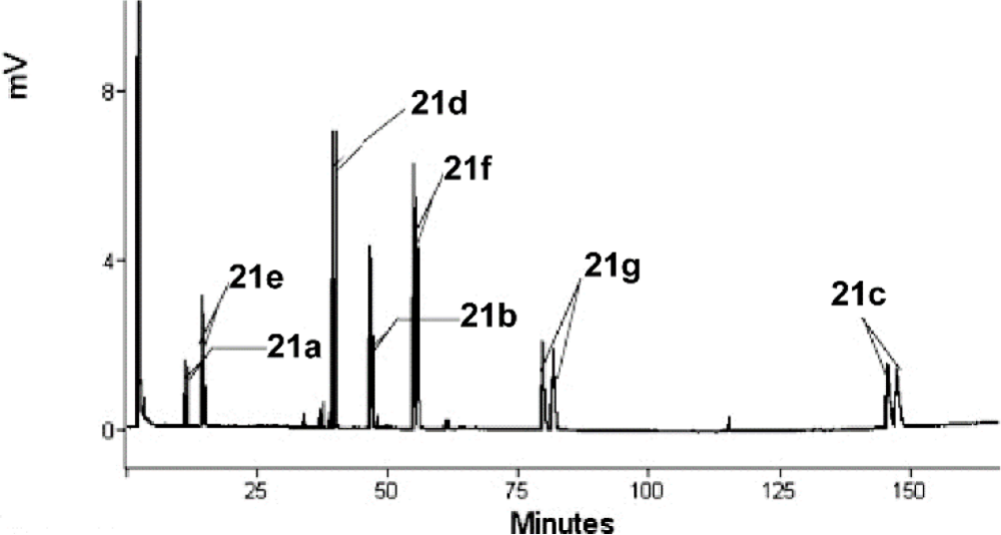
Gas chromatogram of product mixture of reduction products of seven aliphatic ketones. Modified with permission from ref (26). Copyright 2011 Elsevier.

**Scheme 13:**
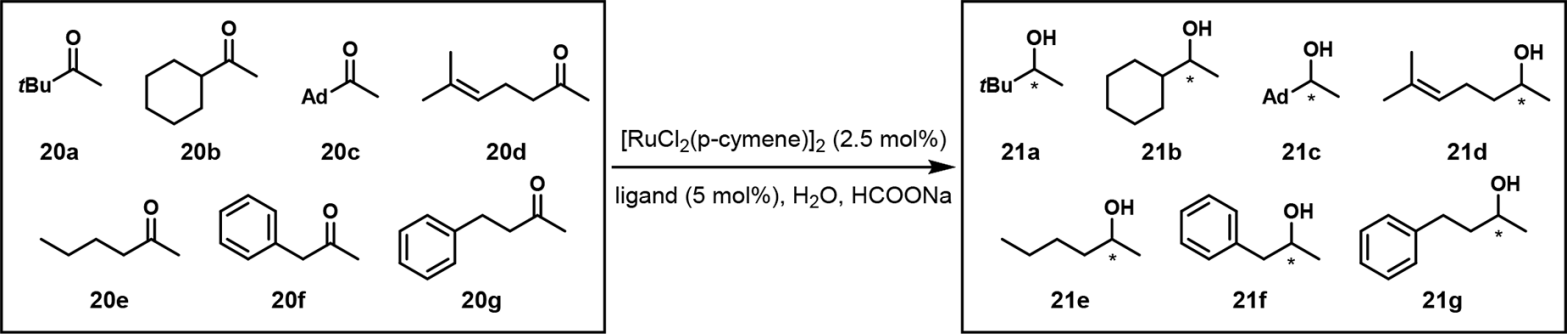
One-Pot Ruthenium-Catalyzed Reduction of Seven Ketones Reaction conditions: ketone mixture (0.5 mmol each), [RuCl_2_(*p*-cymene)]_2_ (0.0875 mmol), ligand (0.175 mmol), HCOONa (10 mmol), H_2_O (4 mL), 30 °C. Ad = 1-adamantyl.

In 2015, the McIndoe group published their study of the relative reactivity of aryl iodides under copper-free Sonogashira coupling conditions. They pooled six aryl iodides **22a**–**f** and acetylene **24** to form coupled products **23a**–**f** (Scheme 14) and analyzed the kinetics by pressurized sample infusion electrospray ionization mass spectrometry (PSI-ESI-MS). This method allowed for real-time monitoring of the intensities of different mass peaks to determine the reaction kinetics. The reaction was monitored until 90% consumption of the phenylacetylene, and the linear kinetic regime of each product formation was used to determine the rate. This was then used to create a Hammett plot, and ρ was 1.4, which indicated the favorability of the *para*-substituted electron-withdrawing groups. Because the purpose of this application of OPMSS was to investigate kinetics, only one catalyst system was used.^27^

**Scheme 14:**
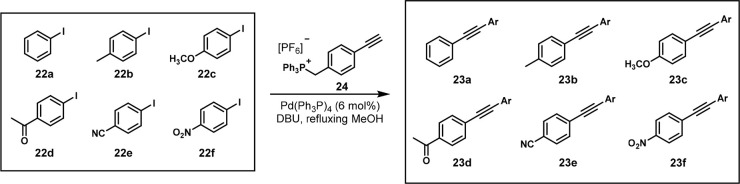
One-Pot Sonogashira Couplings of Six Aryl Iodides with Acetylene **24** with Products Analyzed by PSI-ESI-MS Reaction conditions: mixture of aryl iodides (2 μmol each), **24** (0.012 mmol), Pd(PPh_3_)_4_ (0.72 μmol), DBU (0.12 mmol), MeOH (20 mL). Ar = PhCH_2_PPh_3_.

In 2016, the Flitsch and Barran group reported the biocatalytic PSL lipase enzyme amidation of esters reactions using five substrates in one pot. They used direct infusion ion mobility mass spectrometry (IM-MS) to analyze the products. They used a “twin peak” method involving heavy-isotope labeling of exactly half of each substrate. This resulted in each substrate presenting as one IM peak with two easily recognizable mass peaks, thereby making the identification of a substrate in a complex mixture more facile (Figure 13). Two OPMSS experiments were performed: one screened five esters **25a**–**e** with PSL and piperidine **26a** (1:1 unlabeled/deuterium-labeled), which formed amides **27aa–ea**, and the other screened five amines **26a**–**e** with ester **25a** (1:1 unlabeled/deuterium-labeled), which formed amides **27aa–ae** (Scheme 15). They successfully resolved and identified the substrate peaks. Using **27aa** as a positive control, **27ca** and **27ea** were primarily formed in the ester screen, and in the amine screen, **27ab** and **27ae** were the major products.^28^

**Figure 13:**
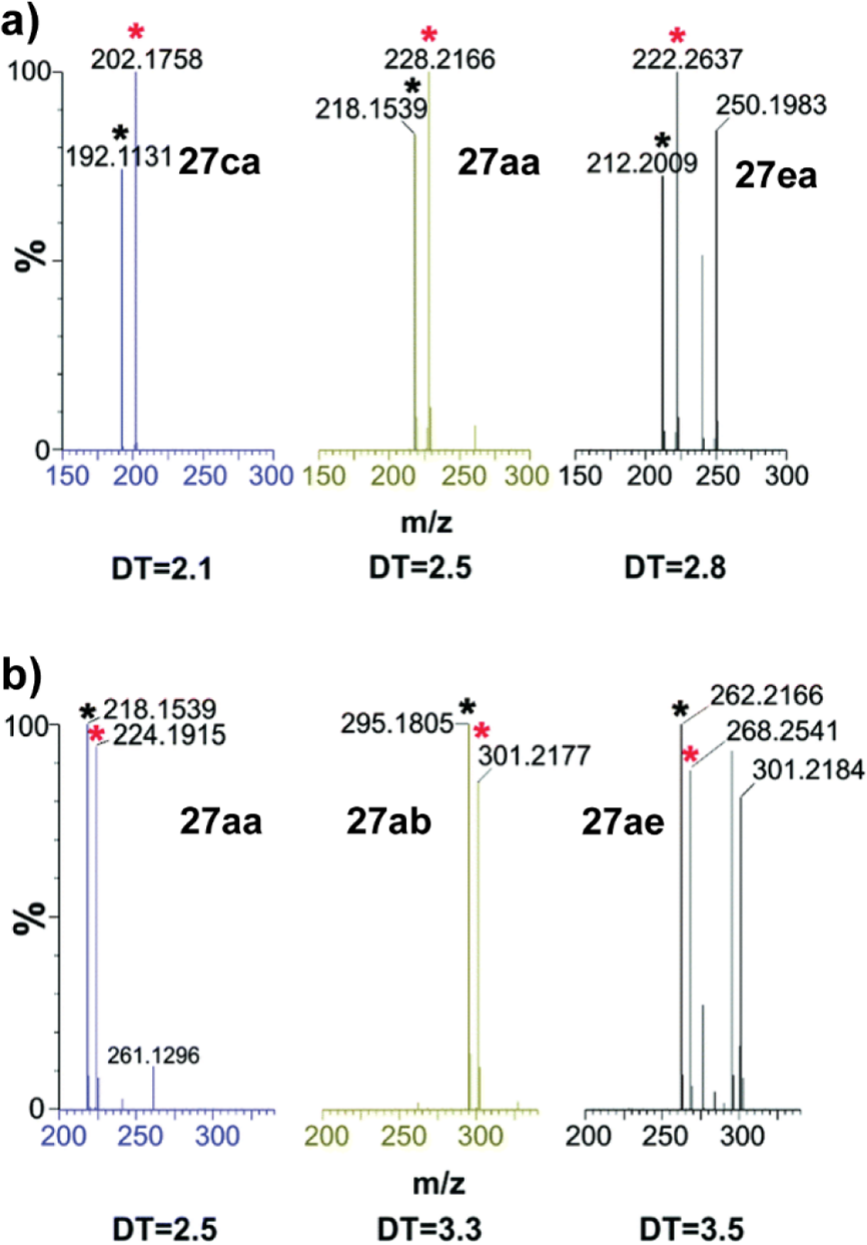
Mass spectra of (a) ester screen and (b) amide screen (DT = drift time). Modified with permission from ref (28). Copyright 2016 Royal Society of Chemistry.

**Scheme 15:**
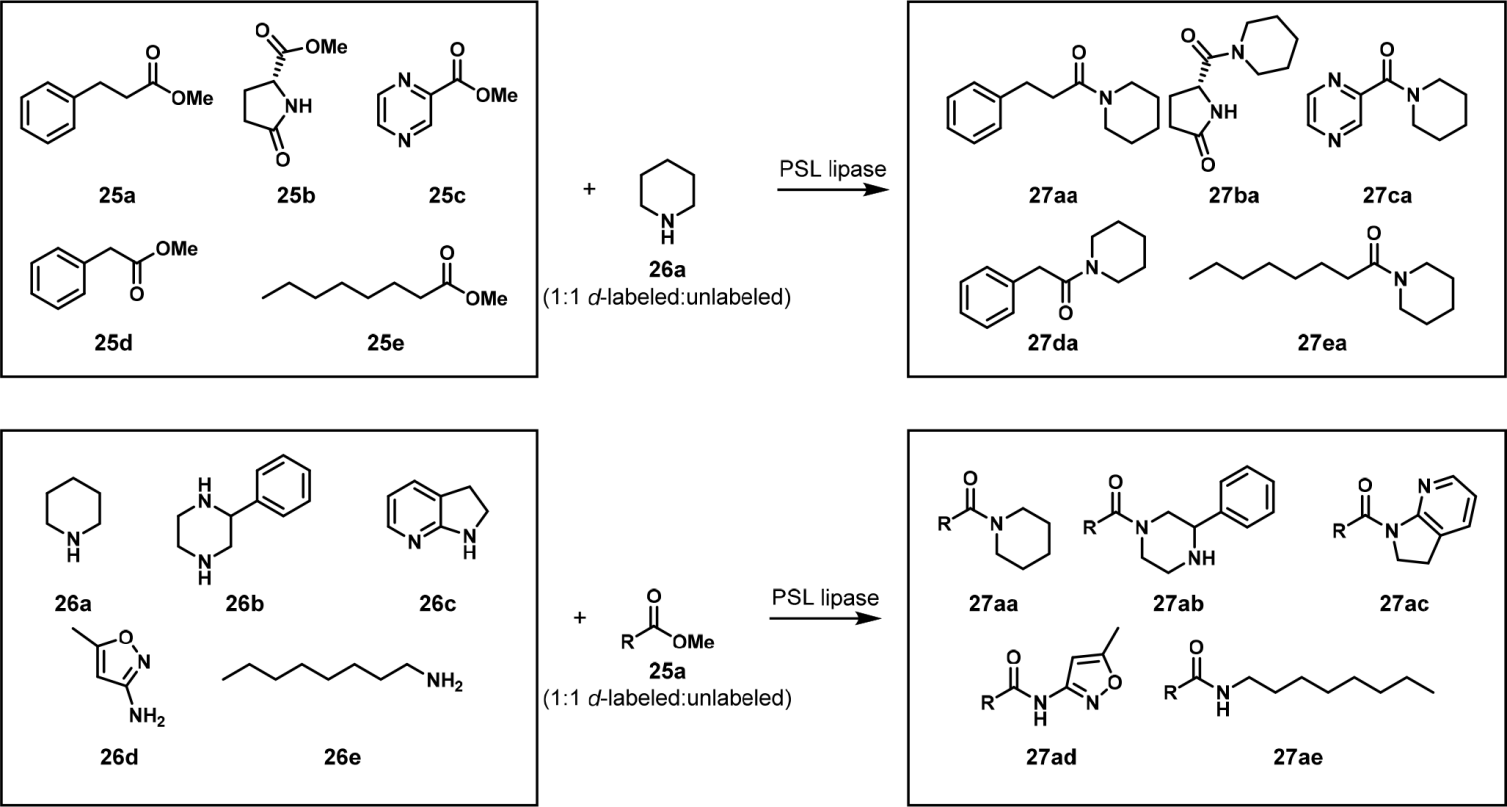
Ester Screen in Which a Mixture of Five Esters Was Coupled with Piperidine and Amine Screen in Which a Mixture of Five Amines Was Coupled with Methyl Ester **25a** The detailed reaction conditions are unavailable. R = CH_2_CH_2_Ph.

In 2016, the O’Neil group disclosed the use of GC × GC to separate 27 compounds (9 substrates and a pair of enantiomers from the 9 substrates after enantioselective reduction). They used this method to test the reduction of nine ketone substrates **14e**, **14i**, **14j**, and **14m**–**r**, investigated by the Li and Gao group,^29^ to their corresponding alcohols **15e**, **15i**, **15j**, and **15m**–**r** in a one-pot fashion (Scheme 16). The ee values were quite similar, and the % conversions followed the same trend between substrates even though they were not always so close to the previously reported single-substrate trials.^30^ This is probably the first example of using GC × GC in OPMSS (Figure 14). The GC × GC technology has excellent potential in OPMSS; however, it has not been used in organic synthesis, possibly because of the scarcity of equipment.

**Scheme 16:**
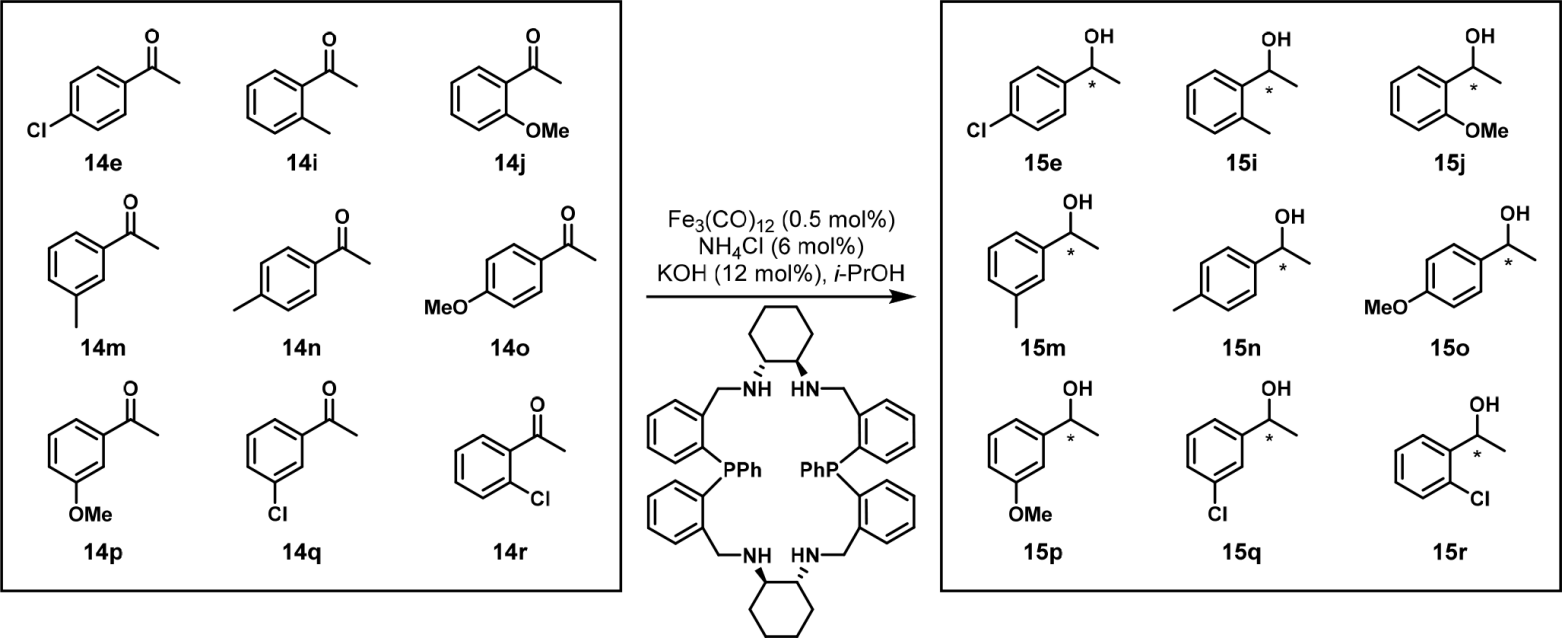
Mixture of Nine Aryl Ketones Enantioselectively Reduced Using an Iron Catalyst and Analyzed by GC × GC Reaction conditions: equimolar mixture of acetophenones (1 mmol total), Fe_3_(CO)_12_ (5 mol %), ligand (5 mol %), NH_4_Cl (6 mol %), NaOH (12 mol %), *i*-PrOH (10 mL), 65 °C, 30 min.

**Figure 14:**
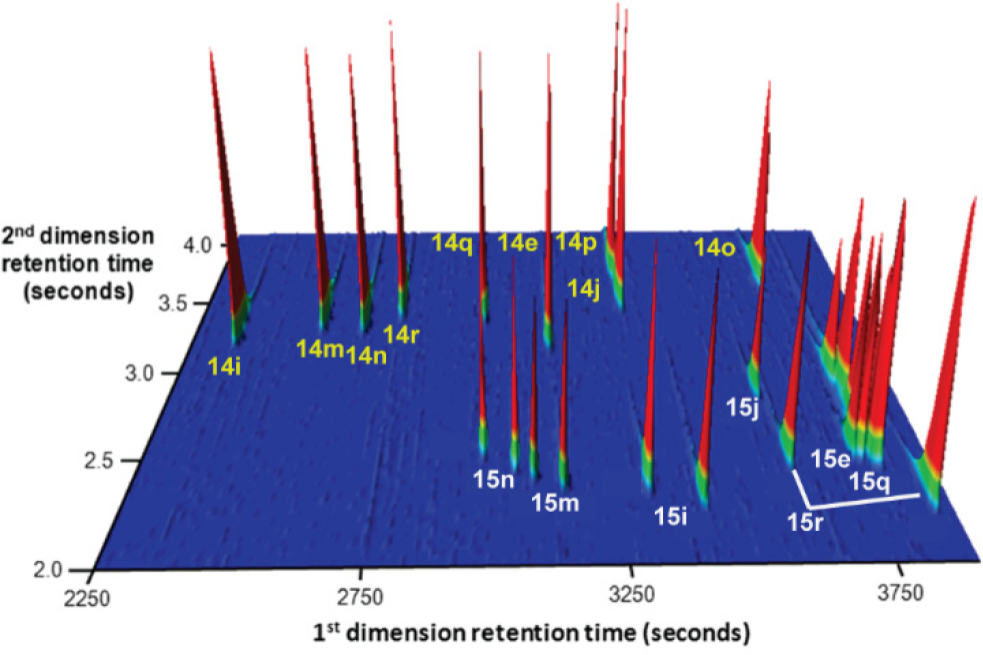
GC × GC chromatogram of 9 ketones and the resulting 18 reduction products. Modified with permission from ref (30). Copyright 2016 American Chemical Society.

In 2019, the List group wrote, “*the multi-substrate screening approach has not previously led to a general and broadly applicable catalyst*.” In light of this, they set out to develop a general catalyst for the enantioselective Diels–Alder reaction between α,β-unsaturated aldehydes with cyclopentadiene. Six enals **28a**–**f** were mixed with cyclopentadiene in one pot to form cyclohexenes **29a**–**f** (Scheme 17), and chiral GC was used to analyze the reaction mixture (Figure 15). They found that imidodiphosphorimidate (IDPi) catalysts provided good reactivity and enantioselectivity, so they used OPMSS to examine multiple IDPi catalysts. Catalysts **30a** and **30b** worked best and provided excellent conversion and enantioselectivity for all enals. They also found that when single-substrate reactions were performed with the pooled enals, the reactivities and enantioselectivities were similar to those of the multisubstrate screens. They then applied this optimized method to many other substrates and observed excellent reactivity and stereoselectivity.^31^

**Scheme 17:**
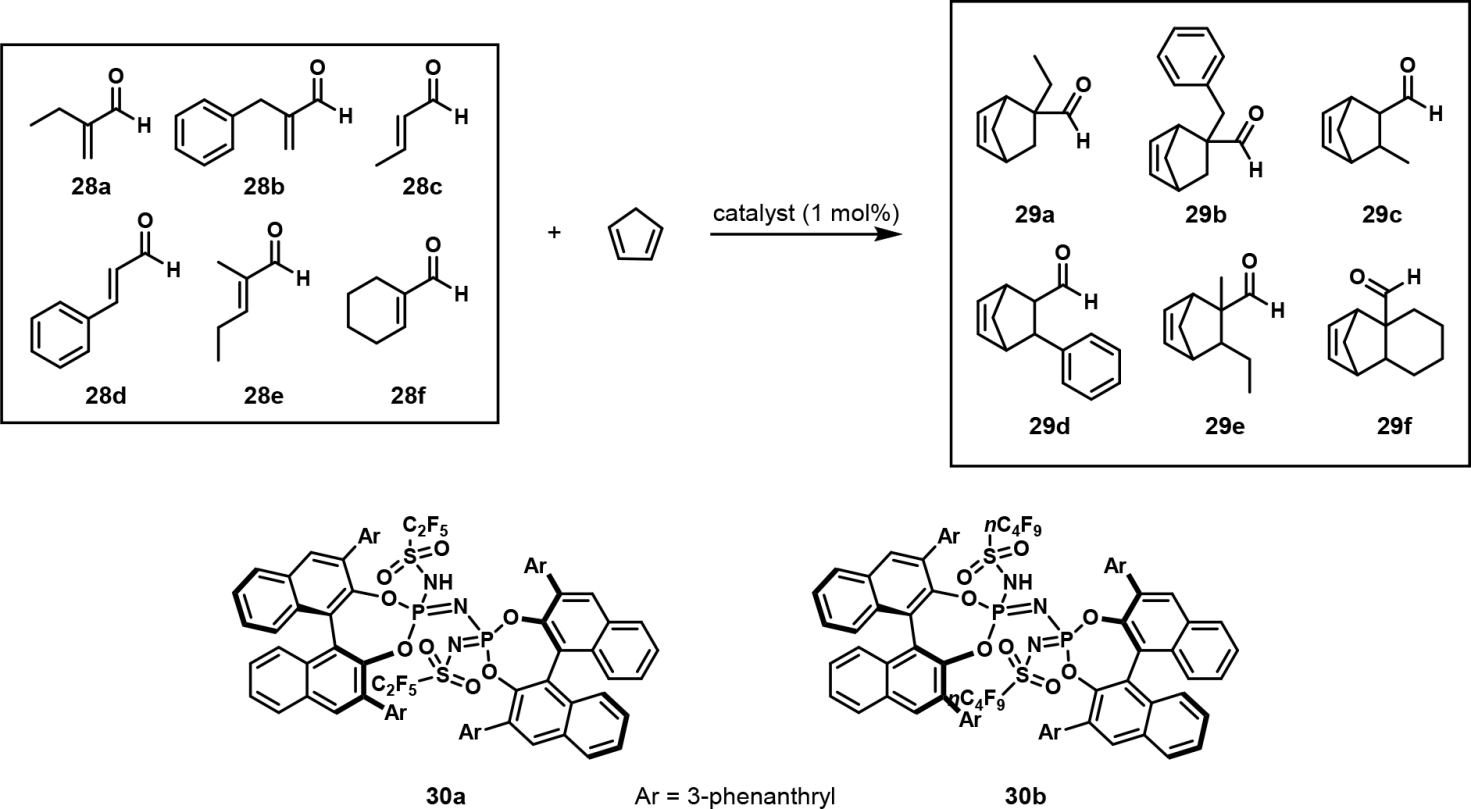
Mixture of Six Enals Reacted with Cyclopentadiene in a Diels-Alder Reaction to Produce Four Stereoisomers of Each Product, with Phosphorimidates **30a** and **30b** as the Most Efficient Catalysts Reaction conditions: mixture of aldehydes (0.02 mmol each), cyclopentadiene (0.6 mmol), catalyst (0.0012 mmol), CH_2_Cl_2_ (0.12 mL), 24 h.

**Figure 15:**
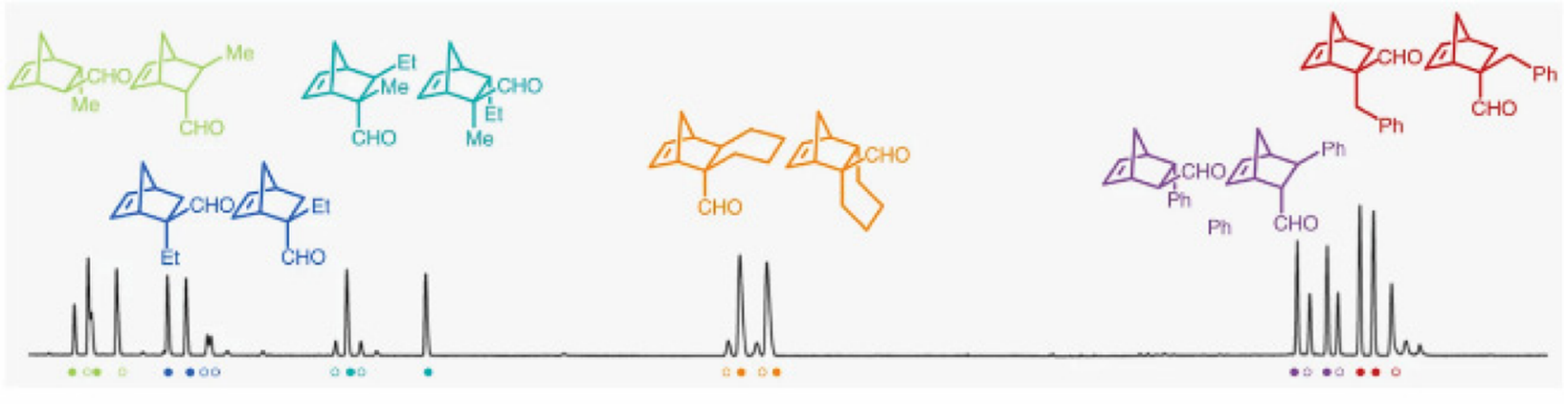
Gas chromatogram of all possible stereoisomers of Diels–Alder reaction between enals and cyclopentadiene. Reproduced with permission under Creative Commons CC-BY 4.0 license from ref (31). Copyright 2019 The Author(s).

In 2021, the List group reported a new type of reaction: adding silyl enol ethers to silyl nitronates enantioselectively. Using a model-substrate approach, they quickly identified IDPi catalyst **30d** as suitable for coupling silyl nitronate **31b** with silyl ketene acetal **33**. However, when **30d** was used with different substrates, the enantioselectivity was much worse. They, therefore, turned to OPMSS to find alternative IDPi catalysts that were effective with more diverse substrates. They pooled silyl nitronates **31a**–**d** and reacted them with **33** to form coupled products **32a**–**d** (Scheme 18). These four substrates were chosen because they had different chemical properties and were separable by chiral HPLC (Figure 16). They found that most of the four substrates had different optimal catalysts, with **30c**, **30d**, and **30e** working best for these substrates. They then expanded the substrate scope by screening new substrates with these three catalysts and could observe good enantioselectivities.^32^ Separate and pooled reactions provided essentially the same yields and ee values.^32^

**Scheme 18:**
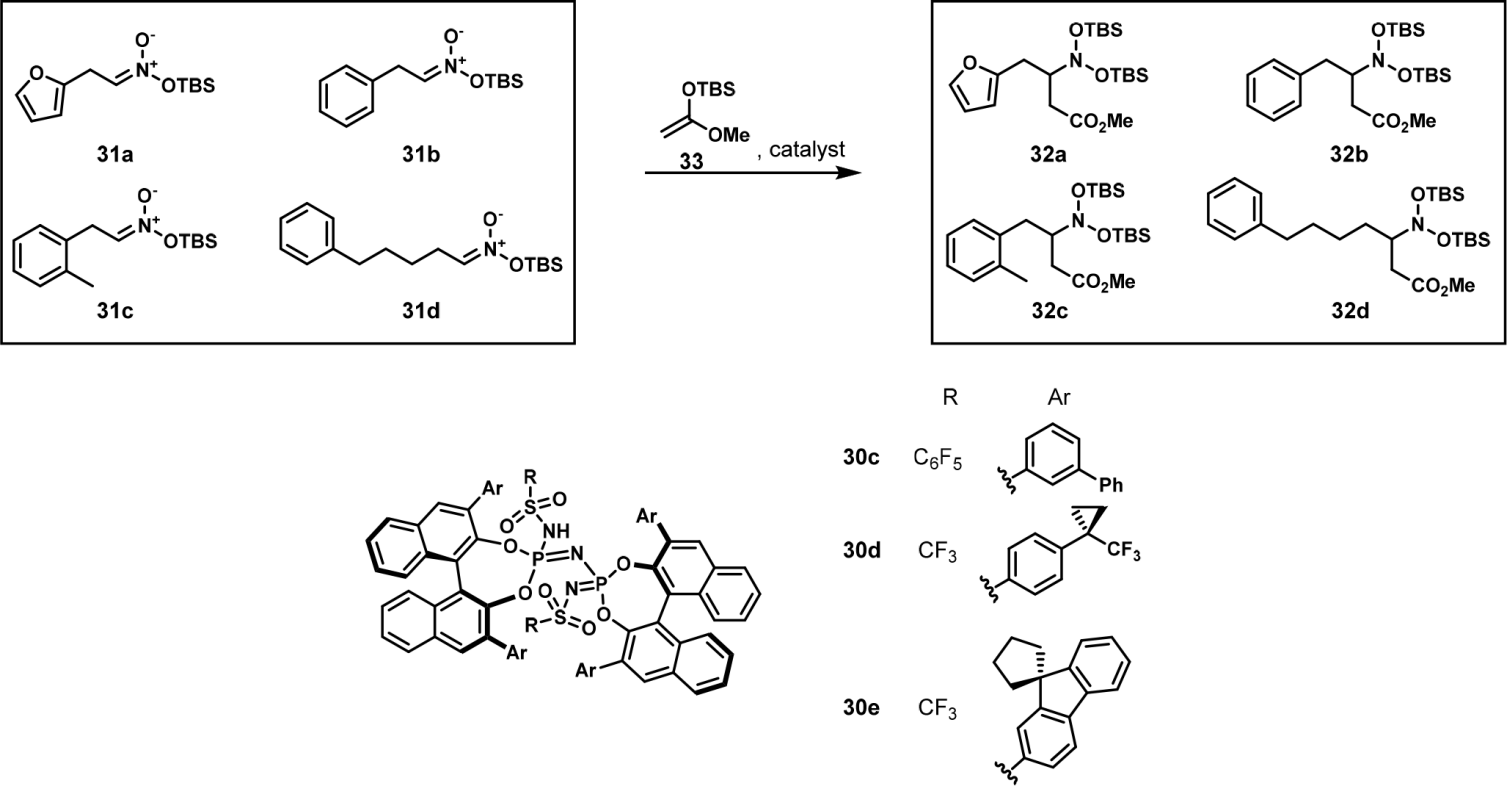
Four Silyl Nitronates Coupled with Silyl Ketene Acetal **33** in One Flask using IDPi Catalysts The detailed reaction conditions are unavailable.

**Figure 16:**
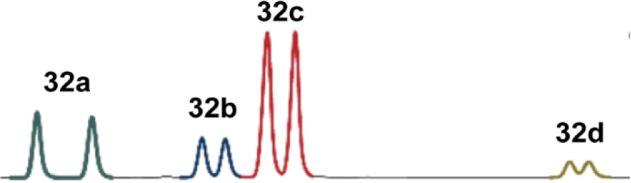
Chiral HPLC chromatogram showing separation of a racemic mixture of four coupling products. Modified with permission under Creative Commons CC-BY 4.0 license from ref (32). Copyright 2021 The Author(s).

In 2021, the Derda group showed the use of a library of fifty-thousand peptide aldehydes with phage display and a biotinylated ylide to perform Wittig reactions (Scheme 19). They carried out a pull-down assay in which the aldehydes that reacted in the Wittig reaction would be biotinylated, the products of which could then be isolated by streptavidin beads. They stopped the reaction after approximately 5% conversion. They then analyzed the results using a deep conversion calculation on the basis of deep sequencing, which calculates the enrichment or decrease of a peptide species to determine the relative initial rates. They selected a series of peptide aldehydes to test individually by HPLC to confirm that the results were consistent with the deep conversion method. Using this generated data set, they discovered the cooperative effect of the first and second N-terminal amino acids and the effect of backbone hydrogen bonding on the reaction rate.^33^

**Scheme 19:**
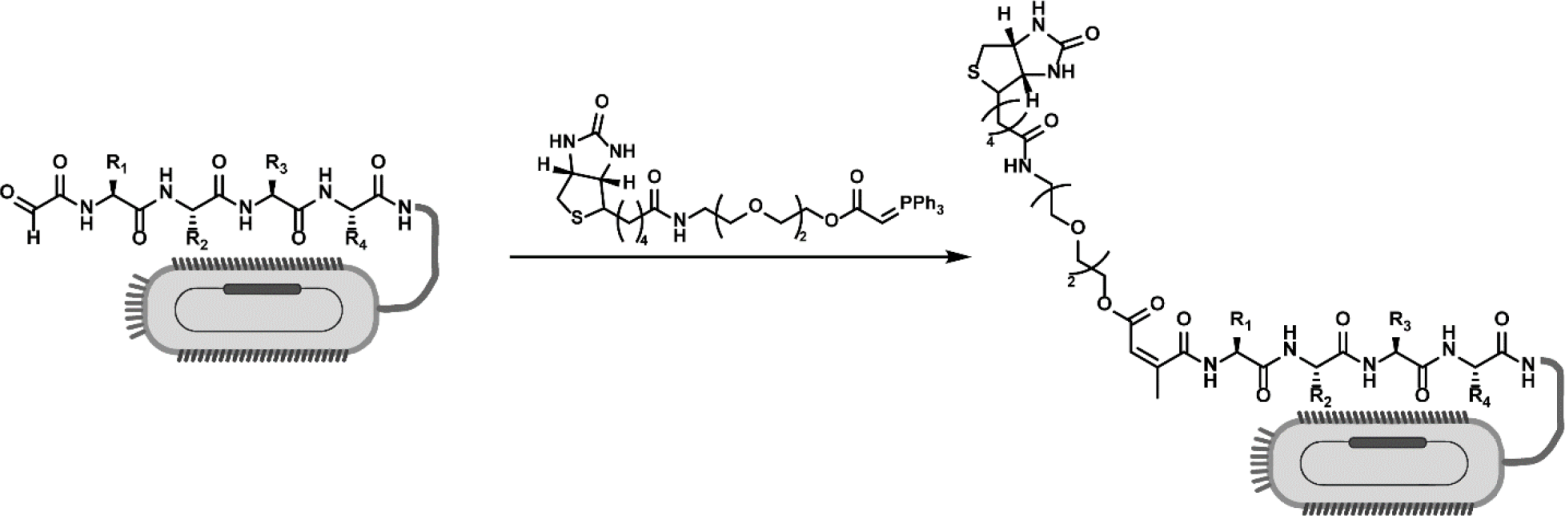
Phage-Displayed Peptide Aldehydes Biotinylated by Wittig Reaction Reaction conditions: 10^12^ pfu (plaque-forming unit) of phage library dissolved in MOPS buffer (100 μL total), NaIO_4_ (1 μL, 6 mM in water), 0 °C, 8 min. Then, methionine (1 μL, 50 mM in water), room temperature, 20 min. Ylide biotin ester (100 μL, 0.8 mM in 200 mM MOPS buffer, room temperature, 10 min).

In 2022, the Jacobsen group reported an enantioselective Pictet–Spengler reaction. They screened 14 aldehyde substrates with 14 different catalysts individually and combinatorially. They then pooled the samples postreaction and analyzed the pooled samples by SFC-MS. Although no catalyst was generally successful for all substrates, they identified catalyst **34** (Figure 17) as the most generally successful using a constructed generality metric (g). They then screened solvents and found that 2-methyl-THF yielded the highest ee values.^34^ Although, by definition, their approach is not OPMSS, their analytical method is closely related. Notably, List et al. stated in the accompanying commentary article that “*the next step should be to refine the method to conduct multiple experiments in a single reaction vessel*.”^35^

**Figure 17:**
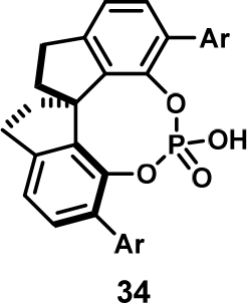
Acid **34** was determined to be the most generally successful catalyst in the Jacobsen group’s enantioselective Pictet−Spengler reaction.

## Future Perspectives

With the advent of machine learning, the generation of thousands of accurate data points from HTE should accelerate discovery and optimization processes in synthetic organic chemistry.^36−39^ However, the required infrastructure is cost-prohibitive in most laboratories. The OPMSS approach is more affordable and may need less time to implement and execute. OPMSS can also rapidly address functional group tolerance in reaction development.^40^ Despite the smaller volume of data obtained through OPMSS, if the data quality is as high as HTE, OPMSS may have potential to be integrated with machine learning.

A caveat for OPMSS is that, as some of the research groups experienced in the above examples, there may be orthogonality issues when combining multiple substrates. Therefore, it is imperative to ensure early in the screening process that the substrates in the system of interest are orthogonal by performing single-substrate reactions and confirming that the results are the same as multisubstrate reactions. Additionally, the substrates and products need to be separable, so it is crucial to ensure that the retention times do not overlap.

Like different ingredients are best for different meals, different reagents or catalysts are likely optimal for different substrates for the same type of reaction. The past examples of OPMSS were reported as part of their efforts to discover the most generally successful reaction conditions. However, because, in some cases, steric factors are dominant, and in others, electronics are more important, it may be more advantageous to use OPMSS to find the optimal conditions for particular substrates more rapidly than the traditional approach.
